# Effect of Sodium Hydroxide Treatments on the Tensile Strength and the Interphase Quality of Hemp Core Fiber-Reinforced Polypropylene Composites

**DOI:** 10.3390/polym9080377

**Published:** 2017-08-19

**Authors:** Romina Del Rey, Ramon Serrat, Jesus Alba, Ildefonso Perez, Pere Mutje, Francesc X. Espinach

**Affiliations:** 1 Universitat Politècnica de València, Centro de Tecnologías Físicas, Escola Politècnica Superior Gandia, Valencia 46730, Spain; jesalba@fis.upv.es; 2 Laboratory of Paper Engineering and Polymer Materials, Dpt. Of Chemical Engineering, University of Girona, Girona 17003, Spain; ramon.serrat@lepamap.udg.edu (R.S.); pere.mutje@udg.edu (P.M.); 3 Departamento de Ciencias Experimentales, Universidad Pablo Olavide, Sevilla 41013, Spain; iperot@upo.es; 4 Design, Development and Product Innovation, Dpt. Of Organization, Business Management and Product Design, University of Girona, Girona 17003, Spain; Francisco.espinach@udg.edu

**Keywords:** hemp core fiber, polypropylene, tensile strength, biocomposites, micro-mechanics, Kelly–Tyson, interphase

## Abstract

The formulation of greener composite materials by substituting glass fibers with natural fibers is a current field of research. If such natural fiber reinforcements come from industrial side streams, as hemp core fibers (HCFs) come from the extraction of hemp strands for the textile industry, an additional advantage can be identified. Nonetheless, such by-product fibers show some drawbacks, such as high lignin contents, which can make it difficult to obtain a good interphase between the fibers and the matrix and to obtain a good fiber individualization. A digestion treatment at different NaOH contents is proposed to eliminate soluble lignin and extractives from the surface of the fibers. At the same time, the use of a coupling agent solves incompatibilities between the fibers and the matrix. The composites were tensile tested and the impact of the proposed treatments is evaluated and discussed. Later, the Kelly-Tyson modified equation and a modified rule of mixtures—the micro-mechanic models—is used to study the impact of such treatments on the quality of the interphase between the polymer and the reinforcement. Both treatments showed a high impact on the tensile strength and the quality of the interphase, obtaining competitive composite materials reinforced with HCFs derived from a by-product.

## 1. Introduction

Environmental concern has constantly increased in the past years. The field of research of composite materials responds to this concern by researching substitutions for materials with high environmental impacts, mainly mineral reinforcements and oil-based matrices [1,2,3,4,5]. In this sense, in the recent years, the literature on the use of natural fibers as a replacement for mineral fibers such as glass-fibers includes a high increase of publications as well as a high increase in reporting on the mechanical properties of the formulated materials [4,6,7,8]. 

**Figure 1:**
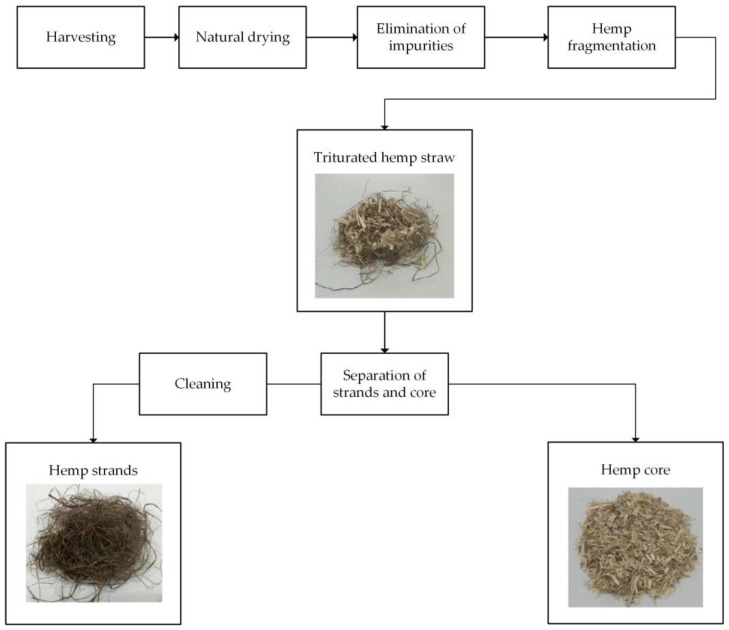
Process used to obtain hemp fibers.

Important factors for choosing a natural source for a reinforcement are its availability, proximity, cost and the need for complementary processes. Then, it is important to bear in mind that there are some natural fibers, such as hemp or abaca, that have higher costs than glass fiber [9,10]. Nonetheless, such fibers show high intrinsic properties, although some processing is needed to obtain fibers with industrial value. The processes used to obtain such fibers produce a quantity of by-products in the form of short, lower-quality fibers. As an example, the process used to obtain hemp fibers is shown in Figure 1 [11,12].

After harvesting, hemp plants are treated to extract the hemp strands that are used in the paper and textile industries. The hemp stems are constituted by 30% to 35% hemp strands, 10% to 15% waste and 50% to 55% hemp core. Once the plants are fragmented, a mixture of hemp strands and hemp core fibers (HCFs) is obtained. Such a mixture is submitted to separation processes, in which HCFs are separated from the hemp strands. Hemp strands are submitted for further cleaning processes to obtain the final high-quality fibers. On the other hand, the hemp core remains a by-product of low value [12].

Hemp core is usually used as bedding for livestock, principally because of its high capacity to adsorb liquids. One of the main drawbacks of hemp core is its high lignin content [12,13]. Nonetheless, there are treatments, such as NaOH pulping, that allow for the obtaining of chemi-thermomechanical pulps with an increased presence of celluloses on their surface and enhanced individualization [14,15,16].

Hemp has already drawn the attention of researchers intending to design more environmentally friendly composites [17,18,19,20], and the studies present promising mechanical properties and the ability of such composites to replace glass fiber-based composites. HCFs have also drawn the attention of researchers, and there is literature on their use as reinforcements for different polymeric matrices [11,12,13,21]. However, to the best knowledge of the authors, this is the first time that HCFs as a by-product have been used as a reinforcement.

In this work, HCFs, obtained as a by-product of the fragmentation of hemp stems to produce hemp strands for the papermaking and textile industries, were used to produce polypropylene (PP)-based composites. Composites containing 40% *w*/*w* HCF were produced and tensile tested. In order to obtain high mechanical properties, the hemp cores were cooked with different percentages of sodium hydroxide (NaOH), and chemi-thermomechanical pulps with a reduced presence of lignin on their surface were obtained. At the same time, different amounts of coupling agent were tested to solve the incompatibilities between the hydrophobic matrix and the hydrophilic reinforcements. The effect of both treatments on the tensile strength of the composites was reported and discussed. Following this, a micro-mechanics analysis was conducted to assess the impact of the mentioned treatments on the quality of the interphase between the HCFs and the PP. A modified rule of mixtures for the tensile strength and the modified Kelly-Tyson equation with the solution provided by Bowyer and Bader were the models used to perform the micro-mechanics study. 

## 2. Materials and Methods 

### 2.1. Materials

The untreated hemp cores of Cannabis Sativa were provided by (Agrofibra S.L., Puigreig, Spain). A PP ISPLEN 090 G2M (Ressol-YPF, Tarragona, Spain) was used as the polymeric matrix. Some composites added Epolene^®^ (Eastman, Middelburg, The Netherlands), a maleic-grafted polypropylene (MAPP), as coupling agent.

The other reactants used included decahydronaphtalene (decalin, ScharLab S.L., Barcelona, Spain) to dissolve the matrix in the fiber extraction during the composite process, and NaOH and anthraquinone (BASF, Tarragona, Spain), used to prepare the fibers. The reactants were used without any further purification.

### 2.2. Hemp Core Treatment

The hemp cores were treated for 90 minutes at 98 ± 2 °C in a 15 L batch reactor. Three different batches containing 5%, 7.5% and 10% NaOH were prepared. In addition to the NaOH, the reactor included 0.1% anthraquinone. The liquid/solid ratio was set at 10. Following this, the digested fibers were washed and dispersed by using a pulp disintegrator. Then, the pulp was defibrated using single-disk Sprout-Waldron equipment. Finally, the fibers were dried for 24 h at 80 °C in a Dycometal oven.

### 2.3. Composite Preparation

The composites with 40% *w*/*w* HCF were prepared in a Gelimat kinetic mixer (model G5S, Draiswerke, Mahaw, New Jersey, USA). The mixture was prepared at 2500 rpm for 2 min until a discharge temperature of 210 °C was achieved. When necessary, as a result of the composite formulation, the coupling agent mixed with the PP was added.

The composites were granulated in a blade mill equipped with 10 mm mesh and were kept in an oven at 80 °C until they were needed, in order to prevent moisture absorption.

### 2.4. Specimen Obtainment

The normalized specimens for the tensile test were injection-molded by means of a Meteor 40 injection machine (Mateu & Solé, Barcelona, Spain). At least 10 test specimens from each composite blend were injection-molded. The processing temperatures of the three heating areas were 175, 175, and 190 °C, the last of these corresponding to the injection nozzle. The first and second pressures were 120 and 37.5 kgf/cm^2^, respectively. The dog bone specimens (approx. 160 × 13.3 × 3.2 mm^3^) were used to measure the tensile properties, in agreement with the ASTM D638.

### 2.5. Mechanical Characterization

According to the ASTM D638 standard, the specimens were stored in a Dycometal conditioning chamber at 23 °C and 50% relative humidity for 48 h prior to the mechanical testing. Following this, the composites were tensile tested in a universal testing machine (InstronTM 1122, Mark-10 Corporation, Copiague, New York, NY, USA) fitted with a 5 kN load cell and operating at a rate of 2 mm/min, according to the ASTM D638 standard. Results were obtained from the averaging of at least five samples.

### 2.6. Fiber Extraction from the Composites

A Soxhlet apparatus was used to extract the reinforcing fibers from the composites by matrix solubilization, using decalin as the solvent. Small parts of the composite were cut, placed inside a cellulose filter and set into the Soxhelt equipment. The fiber extraction lasted for 24 h. The fibers were extracted and rinsed with acetone and then distilled water in order to remove the solvent residue. Finally, the fibers were dried in an oven at 105 °C for 24 h.

### 2.7. Morphologic Analysis of the Fibers

The length and diameter distributions of the extracted fibers were characterized by means of a MorFi Compact instrument (Morfological fiber analyzer, Techpap SAS, Grenoble, France). The equipment measured between 25,000 and 30,000 fibers. Four samples of each type of fiber were analyzed.

## 3. Micro-Mechanics Models

All the micro-mechanics used to model the behavior of the tensile properties of the composites were lineal. The literature endorses the use of such models mainly in the case of good interphases between the reinforcement and the matrix [22,23,24,25]. 

### 3.1. Modified Rule of Mixtures

One of the most simple but elegant ways to model the contribution of the phases to a property of a composite is the use of a rule of mixtures. The literature shows a diversity of formulations for such equations, but all are based on a combination of the contribution of the phases, equalized by their volume fractions and, in some cases, by a number of efficiency factors [26,27,28]. An accepted formulation of a modified rule of mixtures (mRoM) for short-fiber semi-aligned composites is

(1) $$\sigma_{t}^{C}\;=\chi_{1}\cdot \chi_{2}\cdot \sigma_{t}^{F}\cdot V^{F}+\left(1-V^{F}\right)\cdot \sigma_{t}^{M*}$$

where σ_t_^C^ is the ultimate tensile strength of the composite, σ_t_^F^ is the intrinsic tensile strength of the reinforcement, *V*^F^ is the volume fraction of the reinforcement, and σ_t_^M*^ is the contribution of the matrix at the ultimate strain of the composite; χ_1_ and χ_2_ are the orientation and the length factors, respectively, used to equalize the contribution of the semi-aligned short-fiber reinforcement. Usually, a coupling factor *f_c_* is obtained by multiplying such factors (*f*_c_ = χ_1_·χ_2_). It is accepted that well-bonded composites show coupling factors ranging from 0.18 to 0.2 [29,30].

The values for the matrix and the composite are easily acquired experimentally, but the intrinsic tensile strength of the reinforcement and the coupling factor are not so easily acquired. Because of their length, testing the reinforcing fibers is difficult or sometimes impossible. Additionally, there is controversy on the use of experimental values of the fibers to model the behavior of a composite [31]. Therefore, the use of additional models is required to achieve the intrinsic tensile strength of the reinforcement.

### 3.2. Modified Kelly-Tyson Equation

Kelly and Tyson developed a new formulation for the rule of mixtures [32]. These authors divide the reinforcements into sub- and super-critical categories, depending on their lengths. The tensile loads of the composite are transferred from the matrix to the surface of the fibers via shear stress. Then, the stresses are accumulated from both ends of the fiber toward its middle. The longer the fiber, the higher its final accumulated load, up to the moment at which the intrinsic tensile strength of the reinforcement is reached and the reinforcement breaks, which coincides with the critical length [33]. The original Kelly-Tyson equation was formulated for aligned reinforcements, but later on, a modified version adding an orientation factor (χ_1_) was proposed [14,33,34]:

(2) $$\sigma_{t}^{C}\;=\;\chi_{1}\left(X+Y\right)+Z$$

where *X*, *Y* and *Z*, are the contributions of the subcritical fibers, the critical fibers and the matrix, respectively. Then, such contributions are defined by

(3) $$X=\sum_{l=0}^{l=lc}\left[\frac{\tau \cdot l\cdot V_{l}^{F}}{d^{F}}\right]$$

(4) $$Y=\overset{\infty }{\underset{l=lc}{\sum }}\left[\sigma_{t}^{F}\cdot V_{l}^{F}\cdot \left(1-\frac{\sigma_{t}^{F}\cdot d^{F}}{4\cdot \tau \cdot l}\right)\right]$$

(5) $$Z=\left(1-V^{F}\right)\cdot \sigma_{t}^{M*}$$

where τ is the interfacial shear strength that defines the maximum shear load transfers allowed by the interphase between the reinforcement and the matrix; and *d*^F^ and *l* are the diameter and the length of the fibers, respectively. The length is a variable that is defined by the morphological analysis, when a length distribution is obtained and the volume fraction of such a length (*V*_l_^F^) is also defined. The rest of the parameters are already defined for the mRoM (Equation (1)).

In any case, the Kelly-Tyson equation has three unknowns, σ_t_^F^, τ and χ_1_. Fortunately, Bowyer and Bader developed a method to solve the equation [35].

### 3.3. Bowyer and Bader Method

**Figure 2:**
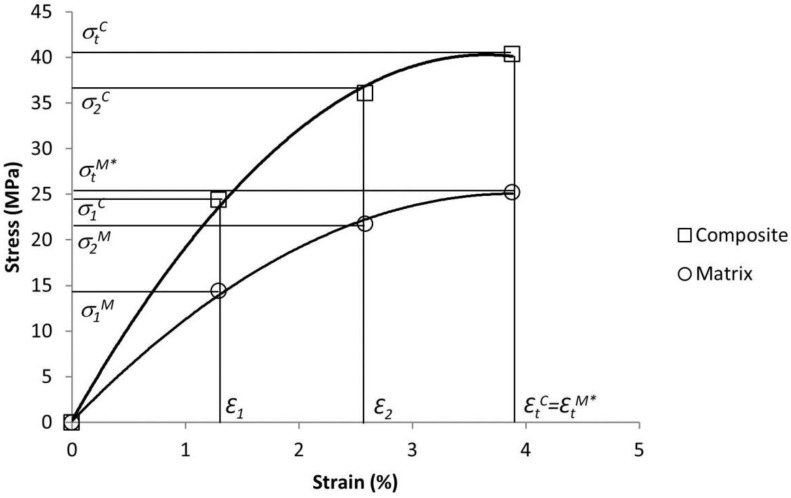
Typical stress-strain curves of a short-fiber semi-aligned reinforced composite and of a polypropylene (PP) matrix. The intermediate strain points used to solve the Kelly-Tyson modified equation are indicated by the 1 and 2 subscripts.

The solution proposed by Bowyer and Bader is based on several assumptions, the most relevant being that the strain of all the phases is the same: Ɛ_t_^M^ = Ɛ_t_^F^ = Ɛ_t_*^C^* (the strains of the matrix, the reinforcement and the composite, respectively). Then, in the elastic section of the stress-strain curve, it is fulfilled that *E*_t_^F^ *=* *Ɛ*_t_^M^*·E*_t_^F^, where *E*_t_^F^ is the intrinsic Young’s modulus of the reinforcement. Bowyer and Bader proposed the use of the experimental data of two strain points (Figure 2). 

Then, Equation 2 can be expressed, for the two intermediate strains, as

(6) $$\sigma_{i}^{C}-\;Z_{i}=\;\chi_{1}\left(X_{i}+Y_{i}\right)$$

The left-hand side of the equation refers to available experimental data; the right-hand side depends on the value of the critical length and is computed by using Equations (4) and (5). Then, the ratio for Equation (6) expressed for the points 1 and 2 is proposed; deleting the orientation factor, we obtain

(7) $$R=\frac{\sigma_{1}^{C}-\;Z_{1}}{\sigma_{2}^{C}-\;Z_{2}};R^{*}=\frac{X_{1}+Y_{1}}{X_{2}+Y_{2}}$$

*R* has a constant value; thus, a value for τ is proposed, and by numerical methods, it is possible to obtain a solution in which R = R^*^.

### 3.4. Hirsch’s Model

As for the intrinsic tensile strength of the fibers, the intrinsic Young’s modulus of short fibers is also difficult to obtain experimentally. Thus, the use of a micro-mechanic equation is proposed. Hirsch’s model is a combination of the Voigt and Reuss models that equalizes the orientation effects of the fibers in the Young’s modulus of the composite [36,37]. The equation can be expressed as

(8) $$E_{t}^{C}=\beta \cdot \left(E_{t}^{F}\cdot V^{F}+E_{t}^{M}\cdot \left(1-V^{F}\right)\right)+\left(1-\beta \right)\frac{E_{t}^{F}\cdot E_{t}^{M}}{E_{t}^{M}\cdot V^{F}+E_{t}^{F}\cdot \left(1-V^{F}\right)}$$

where *E*_t_^C^, *E*_t_^F^, and *E*_t_^M^ are the elastic modulus of the composite, the reinforcement, and the matrix, respectively. The factor β equalizes the parallel and perpendicular models and it has been reported that, for natural fiber composites, a value of β = 0.4 adequately reproduces the results obtained experimentally [38,39].

## 4. Results and Discussion

### 4.1. Hemp Core Fiber Characteristics after NaOH Treatments

The NaOH soft treatments had the objective of cleaning the surface of the fibers form extractives, soluble lignin and insoluble lignin and to ease fiber individualization. The virgin HCFs, as straws and mechanical pulps (0% NaOH), were impossible to properly individualize, showing high percentages of fiber bundles. For the 5% NaOH treatments, it was not possible to obtain properly individualized HCFs. These treatments also had some impact from hemicelluloses mixed with the lignin contents. As a direct consequence of the reduction of the lignin contents in the fiber surfaces, the percentage contents of celluloses and hemicelluloses increased, and as did the availability of OH groups. This was reflected by the Kappa number (Table 1).

The kappa number of the 7.5% NaOH-treated fibers was found to be 7% lower than that of the 5% NaOH-treated sample. The 10% NaOH-treated pulp showed a Kappa number 28% and 19% lower than the 7.5% and 5% NaOH treated pulps, respectively. Thus, the NaOH treatment responded well to the objective of increasing the presence of the OH content at the surface of the fibers [26]. Certainly, the increases in the severity of the NaOH treatments produced decreases in the yield of the process, understood as the amount of treated fibers against the initial biomass. Nevertheless, all the treatments showed yields in the spectrum of chemi-thermomechanical or semi-chemical processes high enough to consider them as being in the low range of the high-yield treatments. These yields ensure the minimum creation of by-products from a by-product, being in line with the principles of green chemistry [40]. Consequently, higher percentages of NaOH were discarded as lower yields were obtained.

The weighted length of the fibers increased with the intensity of the NaOH treatments. This was not surprising because, as the percentages of lignin decreased, the ease of individualizing the fibers increased. Thus, the attrition of the fibers during the individualization process also decreased. The 10% NaOH-treated fibers were 26.3% longer than the 5% NaOH-treated fibers. The diameters of the fibers showed slight variations from one treatment to another. In all the cases, the aspect ratio was higher than 20, ensuring a priori good strengthening capabilities of HCFs. Additionally, the prepared pulps were readily usable in the papermaking industry for the production of brown line papers, that is, fluting and liners.

### 4.2. Impact of NaOH and Coupling Agent Contents on the Tensile Strength of the Composites

Next, composite materials with 40% *w*/*w* HCF were prepared and tensile tested. The obtained properties are shown in Table 2. It was found that the impact of the NaOH treatments of the reinforcements on the tensile strength of the uncoupled composites was modest. 

The tensile strengths of the composites with added 0% MAPP slightly increased in all the cases but remained close the value of the neat matrix. This behavior was likely due to the diffusion of the polymer on the surface of the fibers and due to a mechanical anchoring. Thus, the presence of a higher amount of OH groups on the surface of the fibers did not promote increases of the tensile strength of the uncoupled composites. Similar behaviors are reported in the literature, where, for low amounts of hemp fiber reinforcement, the tensile strength of the composite is lower than that of the matrix [41].

**Figure 3:**
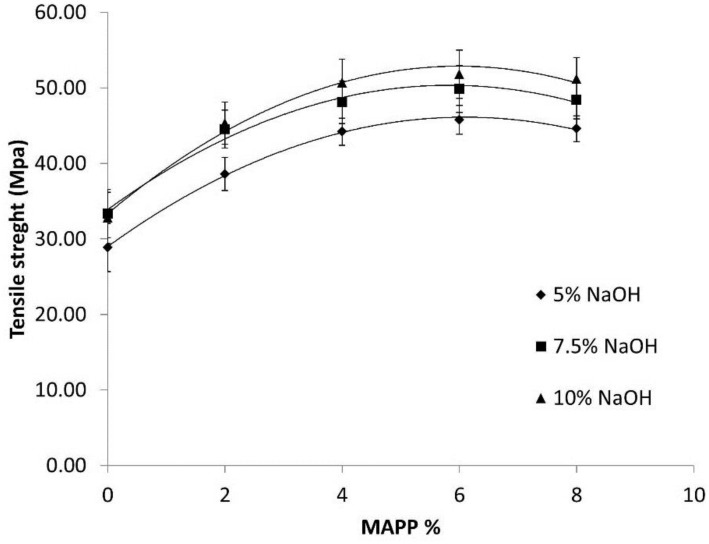
Evolution of the tensile strength of the composites against the coupling-agent contents.

The addition of a 6% MAPP to the matrix slightly changed its properties. Its tensile strength was reduced by 3% and its strain maximum strength increased by 1%. On the other hand, the impact of adding MAPP in the composite formulation was clearly visible (Figure 3).

It is well known and reported in the literature that the different nature of hydrophobic thermoplastics and hydrophilic natural fiber surfaces leads to bad-quality interphases. Thus, chemical modifications can be used to decrease the polarity of the fiber surface. Treatments with acetyls, isocyanates or silanes have been reported as useful and effective but insufficient to provide a good interphase [23]. The use of MAPP as a coupling agent is one of the most recurrent solutions to these problems. While the NaOH treatments allowed for a higher presence of OH groups on the surface of the fibers, MAPP takes further advantage of the presence of such groups. The maleic anhydride groups of the MAPP create hydrogen bonds and covalent ester linkages with the hydroxyl groups of the fiber surface. At the same time, the PP chains of the MAPP entangle with the PP matrix [26,42,43].

As the percentage of MAPP was increased, the tensile strength of the composites, regardless of the amount of NaOH used in the cooking process of the HCFs, increased up to a local maximum identified for the 6% MAPP contents. Higher MAPP contents were derived for slight decreases in the strength of the materials. The literature reports a main cause of such behavior as the coupling agent chains self-entangling instead of reacting with the PP, resulting in slippage [26,44].

The composites with 6% *w*/*w* MAPP clearly showed the combined effect of NaOH treatments and coupling agent addition. These composites, when compared with the matrix, showed tensile strengths with 65%, 80% and 88% increases, for the 5%, 7.5% and 10% NaOH treatments, respectively (Table 2). Compared to the uncoupled composite with the same NaOH treatment, the increases were by 58%, 50% and 57%, respectively. Thus, in both treatments, the NaOH cooking and the addition of coupling agents promoted the increase of the tensile strengths of the composites. Such notable increases indicated the possibility of well-dispersed fibers; however, SEM images are needed to guarantee such a hypothesis.

Regarding the strain at break of the composites (Table 2), an increase that was associated with the presence of MAPP and with the harshness of the NaOH treatment was observed. The composites showed strains at break 3 times lower than those of the matrix but that were usable and comparable to glass fiber-based composites [30].

The work to fracture (*W*_F_) showed that the composite materials increased the ability to endure energy without breaking, while deforming plastically, by the addition of a 6% MAPP and 10% NaOH treatment. The *W*_F_ of such materials was 4.5 times higher than that of an uncoupled composite treated with 5% NaOH.

Other similar natural fiber-reinforced composites, such as chemi-thermomechanical pulp from orange tree prunings treated with 5% NaOH, obtained a tensile strength of 46.5 MPa [16], which was very similar to HCF-based composites (Table 2). The strain at break was the same for the HCF- and the pruning-based composites. By comparison, another by-product, old newspaper-based composites, obtained a tensile strength of 45.2 MPa, which was also very similar to that obtained with HCF-based composites [23].

Therefore, the experimental data show that adding MAPP has a direct effect on the tensile strength of the materials, and a NaOH treatment on fibers makes sense only for coupled composites. This research was followed up to investigate if such conclusions were also valid for the quality of the interphase.

### 4.3. Morphology of the Fibers

As has been mentioned in the modelling section, the Kelly-Tyson model uses the morphology of the fibers as an input to predict the micro-mechanic properties. However, the initial morphologic data of the fibers could not be used (Table 1). It is known that during composite preparation and specimen injection-molding, the reinforcing fibers suffer decreases in their length [33,34,45]. Table 3 shows the morphologic properties of the HCFs extracted from the composite. The weighted and double-weighted lengths were computed and are in agreement with those of the literature [14].

It was found that the length of the fibers, compared with the initial treated pulps, decreased from 50% to 70%. Thus, the aspect ratios decreased in parallel but remained higher than 10%. The diameter of the fibers decreased slightly but remained almost equal.

**Figure 4:**
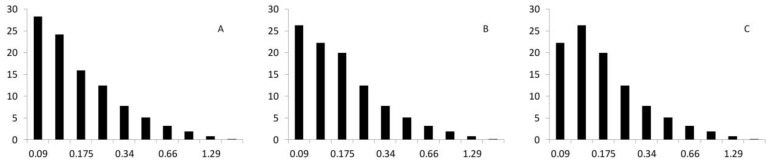
Fiber length distributions. (**A**–**C**) correspond to the 5%, 7.55% and 10% NaOH treatments, respectively.

Figure 4 shows the fiber length distributions for the HCFs extracted from the composite.

It was found that all the distributions were similar and had slight changes in the percentages of short fibers, indicating a higher ratio of fiber shortening in the case of the fibers treated with milder NaOH percentages. It was explained in the introduction to the Kelly-Tyson equation that the fibers were divided into subcritical and supercritical sets. The equation defining the critical length is

(9) $$l_{c}=\frac{d^{F}\cdot \sigma_{t}^{F}}{2\cdot \tau }$$

The diameter of the fibers and the intrinsic tensile strength of the fibers are quasi-invariants because they depend on the phase. Therefore, the interfacial shear strength (τ) defines the critical length that decreases with higher τ values. Thus, the composites with a higher quality interphase are able to take advantage of a higher amount of supercritical fibers. Hence, the 10% NaOH-treated HCF had a slight advantage, as shown by higher mean lengths.

### 4.4. Micro-Mechanics

The micro-mechanic analysis had the objective of assessing the influence of the properties of the phases and their contents on the properties of the composites. Table 4 shows the initial data used to solve the Kelly-Tyson modified equation with the solution provided by Bowyer and Bader. 

The contribution of the matrix was computed by approximating the stress-strain curve of the matrix to a 4th degree polynomial:

(10) $$\sigma_{t}^{M*}=-0.0159\cdot {\left({\varepsilon_{t}}^{C}\right)}^{4}+0.3712\cdot {\left({\varepsilon_{t}}^{C}\right)}^{3}-3.3674\cdot {\left({\varepsilon_{t}}^{C}\right)}^{2}+14.8953\cdot {\varepsilon_{t}}^{C}\cdot 0.0493$$

**Figure 5:**
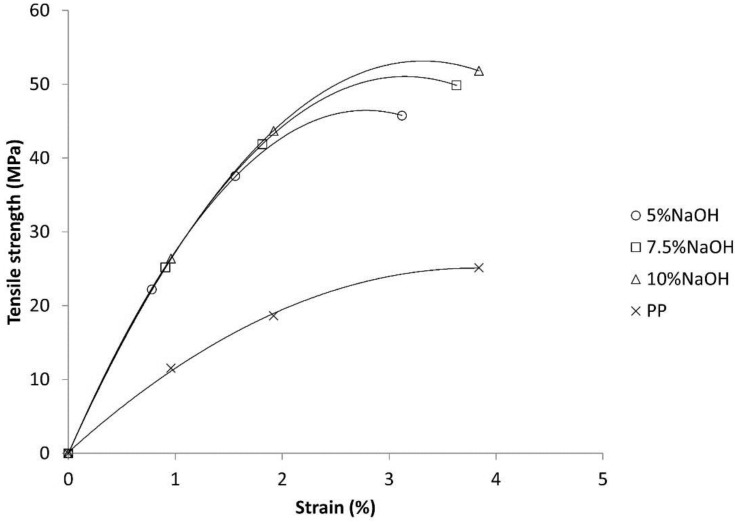
Mean stress-strain curves of the composites and the matrix. The intermediate strain points used to solve the Kelly-Tyson equation are marked above the corresponding curve.

The intrinsic Young’s modulus of the composites was computed by using the Hirsch model (Equation 8). Figure 5 shows the experimental strength and strains, final and intermediate, of the composites and the matrix, used to solve the Kelly-Tyson equation. The Young’s moduli of the composites were very similar: 4.7, 4.7 and 4.8 GPa for the 5%, 7.5% and 10% NaOH-treated composites, respectively. This is clearly shown by the stress-strain curves of the composites, which show few deviations on their initial section slope.

The composites did not show a clearly defined elastic region, but it could be observed that the intermediate points were positioned at the almost-linear sector. The intermediate strain locations were defined as 1/3 and 2/3 of the strain at break of the composites. Once the solution provided by Bowyer and Bader was implemented, the results shown in Table 5 were obtained.

It was found that the orientation factors coincided at 0.29. Such a value is in line with those obtained in the past and already published in the literature [14,22,33,46]. Such literature shows that the value of the orientation factor depends, among other parameters, on the equipment used to injection-mold the specimens [47]. In the cited bibliography, the orientation factor was always found to be inside the range from 0.25 to 0.35.

The interfacial shear strength, as a measure of the quality of the interphase, increased with the intensity of the NaOH treatments. All the values were near that defined by the von Mises criteria (15.9 MPa). If such a value is considered as an upper bound for the theoretical values of τ, then all the interphases could be considered as very good to almost optimal [33].

As for the rest of the parameters, the intrinsic tensile strength of the HCFs increased with the intensity of the NaOH treatment, indicating a better exploitation of the strengthening abilities of these reinforcements. The values were in line with those of the literature, where similar reinforcements obtained intrinsic tensile strengths in the range from 550 to 900 MPa [9,21]. It must be taken in account that the higher values corresponded to high-quality hemp fibers.

**Figure 6:**
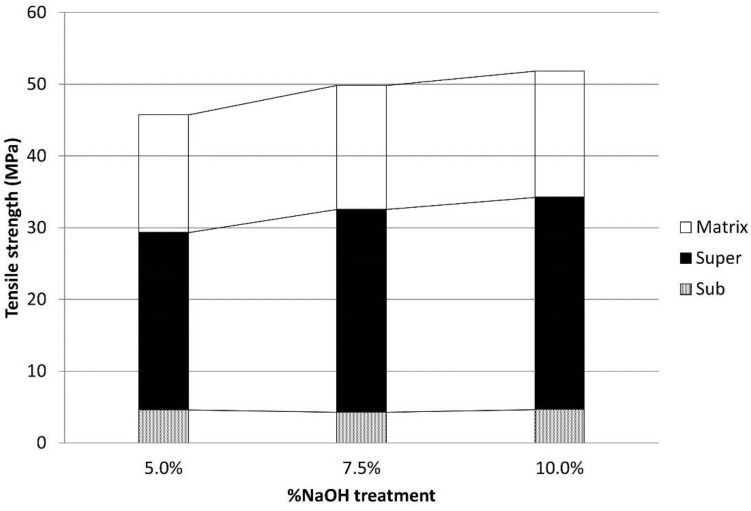
Contributions of the subcritical and supercritical fibers and the matrix to the tensile strength of the composite.

As it was abovementioned, the critical length was computed with Equation (9). The increases of the critical lengths of the HCFs against the NaOH treatments were highly defined by the quality of the interphase and by the higher values of the intrinsic tensile strengths. This was of special importance when applying the Kelly-Tyson modified equation. As can be seen in Figure 4, the fiber length distributions are presented as discrete sets and not as continuous curves. Thus, depending on the value of the critical length, a complete set of fibers could pass from supercritical to subcritical, altering the results. Figure 6 shows the cumulative contribution of the subcritical and supercritical fibers and the matrix to the tensile strength of the composite.

It was found that the main contributions were obtained for the supercritical fibers in all the cases, followed by the matrix. In all the cases, the contribution of the subcritical fibers was modest. Table 6 shows the percentage contributions. It was found that the contribution of the supercritical fibers amounted to more than 50%, the matrix amounted to around 34%, and the subcritical fibers amounted to around 8%.

With the objective of verifying the obtained values, the mRoM (Equation (1)) was solved. The value of the intrinsic tensile strength was then obtained as in Table 5, and the rest of the values are shown in Table 2 and Table 4. Then, the values of the coupling factor were obtained. The equation was also solved for the uncoupled composites (Table 7).

As expected, the coupling factors of the composites when adding 6% MAPP lead to a coupling factor of around 0.2, indicative of good interphases. On the other hand, the uncoupled composites delivered coupling factors of around 0.1, 50% less than the coupled factors. 

Using the mRoM, a correlation between the intensity of the NaOH treatment and the quality of the interphase was not observed, but the solution was highly ballasted by the higher intrinsic tensile strength values.

## 5. Conclusions

Biocomposites with hemp core by-product fiber-reinforced PP were prepared and tensile tested. The hemp core fibers were submitted to a cooking process under 5%, 7.5% and 10% NaOH contents. To the composites were added percentages of a coupling agent ranging from 0% to 8%.

The coupling agent contents had a direct and visible impact on the tensile strength of the composites, solving the incompatibility between the hydrophobic matrix and the hydrophilic fibers. Higher tensile strengths were found for the composites when 6% *w*/*w* MAPP was added.

The cooking of the fibers showed little effect on the tensile strength of the composites without a coupling agent, increasing the value only slightly. Nonetheless, when percentages of MAPP were added to the composites, the materials reinforced with the fibers and treated with the most intensive NaOH process showed noticeably higher tensile strengths. Hence, a combination of the coupling agent and intensive cooking of the fibers had a noticeable impact on the tensile strength of the composites. It was possible to obtain competitive natural fiber by-product-reinforced composites, thus adding value to hemp core fibers. The preparation of the chemi-thermomechanical hemp core fibers produced low percentages of new by-products, which was in line with the principles of green chemistry.

The cooking process stimulated the individualization of the fibers, resulting in longer fibers as the percentages of NaOH used during the preparation of the reinforcements were increased.

The Kelly-Tyson model showed a high impact of the presence of MAPP and the treatment of the fibers on the quality of the interphase between the fibers and the matrix. The more intensive the NaOH treatment, the higher the quality of the interphase. The mRoM was unable to bring about increases in the quality of the interphase against the harshness of the NaOH treatment. The quality of the interphase of the uncoupled composites was half of those coupled with 6% *w*/*w* MAPP. 

The notable increases in the tensile strength of the coupled composites indicated a good dispersion of the fibers in the composite, but more research is needed to guarantee such a hypothesis. A good dispersion of the reinforcement is one of the main parameters for ensuring good tensile properties. 

In the future, obtaining suitable HCFs by using a higher yielding process may be of interest, thus minimizing the creation of by-products and further aligning with the principles of green chemistry.

## Figures and Tables

**Table 1:** Hemp core by-product NaOH-treated fiber experimental parameters.

NaOH	Yield (%)	Kappa	*l*_w_^F^ (µm)	*d*^F^ (µm)	*l*_w_^F^/*d*^F^
5.0%	78.1	73.2	569	23.8	23.9
7.5%	76.4	68.1	689	24.7	27.9
10.0%	66.9	57.0	719	24.6	29.2

**Table 2:** Tensile strength, strain at break and work to fracture of the hemp core fiber (HCF)-reinforced polypropylene (PP) composites.

Composite Formulation			0% MAPP			6% MAPP		
*V*^F^ (%)	HCF (wt%)	NaOH (wt%)	σ_t_^C^ (MPa)	Ɛ_t_^C^ (%)	*W*_F_ (MJ/m^3^)	σ_t_^C^ (MPa)	Ɛ_t_^C^ (%)	*W*_F_ (MJ/m^3^)
0	0	-	27.6 ± 0.50	9.3^*^ ± 0.20	158.51 ± 9.13	26.8 ± 0.20	9.2^*^ ± 0.20	-
0.301	40	5.0	28.86 ± 1.05	1.64 ± 0.16	10.46 ± 0.78	45.77 ± 1.23	3.12 ± 0.23	30.78 ± 1.16
0.301	40	7.5	33.34 ± 0.86	2.13 ± 0.23	15.54 ± 1.01	49.86 ± 1.86	3.63 ± 0.26	40.60 ± 2.39
0.301	40	10.0	32.81 ± 0.50	2.43 ± 0.31	17.68 ± 1.57	51.83 ± 0.50	3.84 ± 0.35	46.75 ± 3.48

^*^The strain for the matrix was measured at the point of maximum strength.

**Table 3:** Morphologic properties of the fibers extracted from the composites.

NaOH	*d*^F^ (µm)	*l*_a_^F^ (µm)	*l*_w_^F^ (µm)	*l*_ww_^F^ (µm)
5%	23.7	207	380	655
7.5%	24.5	213	386	665
10%	24.6	221	397	684

**Table 4:** Data used to solve the Kelly-Tyson modified equation with the method supplied by Bowyer and Bader.

Property	Unit	5.0%	7.5%	10.0%
*E*_t_^F^	(GPa)	25.7	26.3	27.6
σ_t_^M*^	(MPa)	23.5	24.7	25.2
Ɛ_1_	(%)	0.8	0.9	1.0
σ_1_^M^	(MPa)	9.8	11.1	11.6
σ_1_^C^	(MPa)	22.1	25.2	27.2
Ɛ_2_	(%)	1.6	1.8	1.9
σ_2_^M^	(MPa)	16.4	18.0	18.6
σ_2_^C^	(MPa)	37.6	41.9	44.7

**Table 5:** Micro-mechanic properties of the reinforcing fibers and the interphase.

NaOH	χ_1_	τ	σ_t_^F^	*L*_c_^F^
5.0%	0.29	14.03	472	398.7
7.5%	0.29	14.73	548	455.8
10.0%	0.29	15.06	584	477.1

**Table 6:** Percentage contribution of the phases to the tensile strength of the composite.

NaOH	5.0%	7.5%	10.0%
Subcritical	10.1%	8.6%	9.0%
Supercritical	54.0%	56.7%	57.1%
Matrix	35.9%	34.7%	33.9%

**Table 7:** Coupling factors obtained by solving a modified rule of mixtures for the tensile strength of the composites.

NaOH	0% MAPP	6% MAPP
5.0%	0.106	0.206
7.5%	0.118	0.197
10.0%	0.102	0.193
